# Machine learning potential for modelling dynamic hydrogen bond networks in MOF MIL-120

**DOI:** 10.1039/d5sc09058j

**Published:** 2026-04-02

**Authors:** Xin Jin, Yutao Li, Kelian Gaedecke, Xiaoqi Zhang, Berend Smit

**Affiliations:** a Laboratory of Molecular Simulation (LSMO), Institut des Sciences et Ingénierie Chimiques, École Polytechnique Fédérale de Lausanne (EPFL) Switzerland berend.smit@epfl.ch

## Abstract

Metal–organic frameworks (MOFs) are porous materials with the potential for gas adsorption and separation technologies due to their tunable structural and chemical characteristics. However, simulating gas adsorption isotherms in MOFs with DFT-level accuracy remains a key challenge. In this work, we use MIL-120 as a case study and propose a comprehensive computational workflow to fine-tune a pre-trained MACE potential, enabling the creation of accurate machine-learning interatomic potentials tailored for frameworks with dynamic structural behavior. Using this ML potential to accelerate sampling, we uncover a strong coupling between CO_2_ adsorption and the complex dynamic hydrogen-bond network on the MIL-120 pore surface. The presence of CO_2_ induces local configurational rearrangements that reshape the pore environment. This insight provides a generalizable strategy for simulating adsorption in other flexible MOF systems.

## Abstract 2

Metal–organic frameworks (MOFs) are porous materials with the potential for gas adsorption. This work presents a generalizable framework for simulating adsorption in flexible MOFs using machine learning potentials.

## Graphical abstract

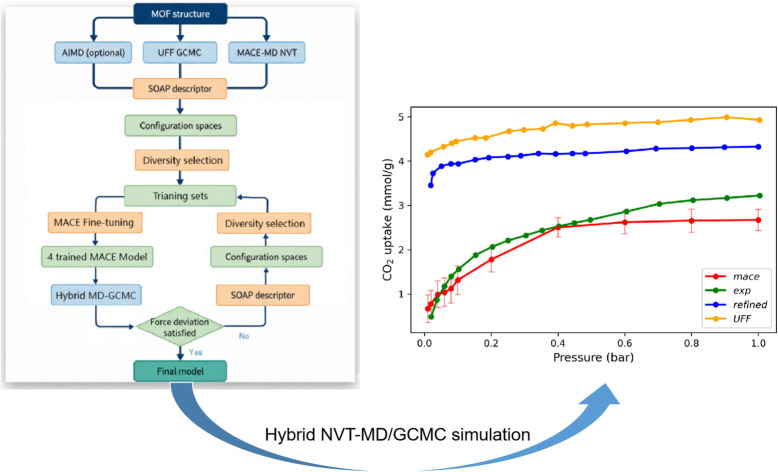

## Introduction

Metal–organic frameworks (MOFs) are porous materials with potential applications in gas adsorption and separation technologies due to their tunable pores and chemical characteristics.^1^ Each year, a large number of MOF structures are designed and synthesized,^2^ and many of them have been experimentally validated to exhibit excellent performance in carbon dioxide adsorption.^3^ High-throughput computational screening methods have been used to identify promising candidates.^4^ Gas adsorption simulation techniques have become highly developed, including the calculation of adsorption enthalpies, Henry coefficients, and isotherm predictions based on Grand Canonical Monte Carlo (GCMC) simulations.^5–7^ These methods typically rely on generic force fields, such as the Universal Force Field (UFF) or Dreiding, to describe the interactions between the adsorbed gas molecules and the framework atoms of the MOFs.

Although classical force field MC and MD simulations have already been widely employed to explore the adsorption and diffusion of guest molecules in MOFs,^6^ inaccuracies resulting from these generic force field can lead to both qualitative and quantitative inconsistencies in the prediction of properties, especially in the case of complex physical and chemical adsorptions involving open-metal sites, parallel aromatic rings, and dynamic hydrogen bonds networks.^8,9^*Ab initio* methods, such as Density Functional Theory (DFT), offer a successful approach for precisely determining complex interaction forces. However, considering the time and computational cost, it is impractical to perform *ab initio* Monte Carlo (AIMC) or Molecular Dynamics (AIMD) calculations for large-scale metal–organic frameworks (MOFs) due to the high computational demands.

Atomistic simulation with machine learning potentials has emerged as a promising approach, offering, at the expense of a modest decrease in computational efficiency compared with force field method, an accuracy comparable to density functional theory. Among these, DeepMD^10^ and NequIP^11^ are mature and promising options for training the machine learning potential of MOFs from scratch. Many works have been reported using machine learning potentials trained from scratch for specific MOFs, including simulations of their gas adsorption properties. For example, Achar *et al.*^12^ developed an ML potential for CO_2_ adsorption in UiO-66, achieving DFT-level binding energies. Similarly, Zheng *et al.*^13^ employed the DeepMD approach to train an ML potential to describe the CO_2_ adsorption in MOF-74-Mg. Goeminne *et al.*^14^ employed the NequIP method for the same system, further improving the accuracy to a level suitable for grand canonical Monte Carlo simulations. In addition to these system-specific models, there are also generalized MOF ML potentials, such as that reported by Yue *et al.*,^15^ who trained a NequIP potential for 8000 Zn-based empty MOFs, which can be used for geometric optimization. These studies generate the dataset for training the potential through time-consuming *ab initio* molecular dynamics simulations. And, to ensure a stable and reliable potential, introduce subsequent active learning cycles. These cycles iteratively refine the potential energy surface. One of the challenges with this approach is that it has limited extrapolation capacity.

Among those machine learning potential methods, MACE, a message-passing model based on Atomic Cluster Expansion (ACE),^16^ uses a different approach. Unlike traditional local potential models, the MACE potential can capture information beyond the strictly local atomic environment, owing to message passing across multiple predefined cutoff ranges within the model, ensuring that MACE is not limited to structural information within a fixed local neighborhood. Instead, it enhances the representation of high-order many-body interactions, thereby improving the accuracy of descriptions in complex atomic environments. More importantly, MACE provides a set of general pre-trained models derived from a wide range of solid-state material systems.^17^ These models allow users to efficiently generate representative machine learning potentials by fine-tuning with a small number of key configurations. Lim *et al.*^18^ performed continuous sampling of CO_2_ at different positions inside MOF pores, and fine-tuned a pretrained MACE potential to obtain a generalized potential for CO_2_ adsorption in rigid MOFs. This approach is expected to yield accurate results for MOFs with well-defined, known binding sites. However, It is still far from providing accurate adsorption energies in all MOFs, let alone isotherms.

Moreover, there is an even more challenging task for simulating the properties of flexible frameworks. In most cases, MOF structures are treated as rigid frameworks, with their structures assumed to remain unchanged during Monte Carlo or Molecular Dynamics simulations. However, this assumption does not hold for all MOFs. In fact, an increasing number of flexible MOF structures have been reported, characterized by pore sizes or surface morphologies that change in response to variations in temperature or pressure.^19,20^ Since gas adsorption occurs at the pore surfaces, the dynamic structural changes of flexible MOFs can have a significant impact on adsorption behavior and therefore cannot be neglected in simulation studies.^21^ It is important to build a potential energy surface (PES) to describe how atoms interact for MC or MD simulations at the DFT level with flexible frameworks.

**Fig. 1:**
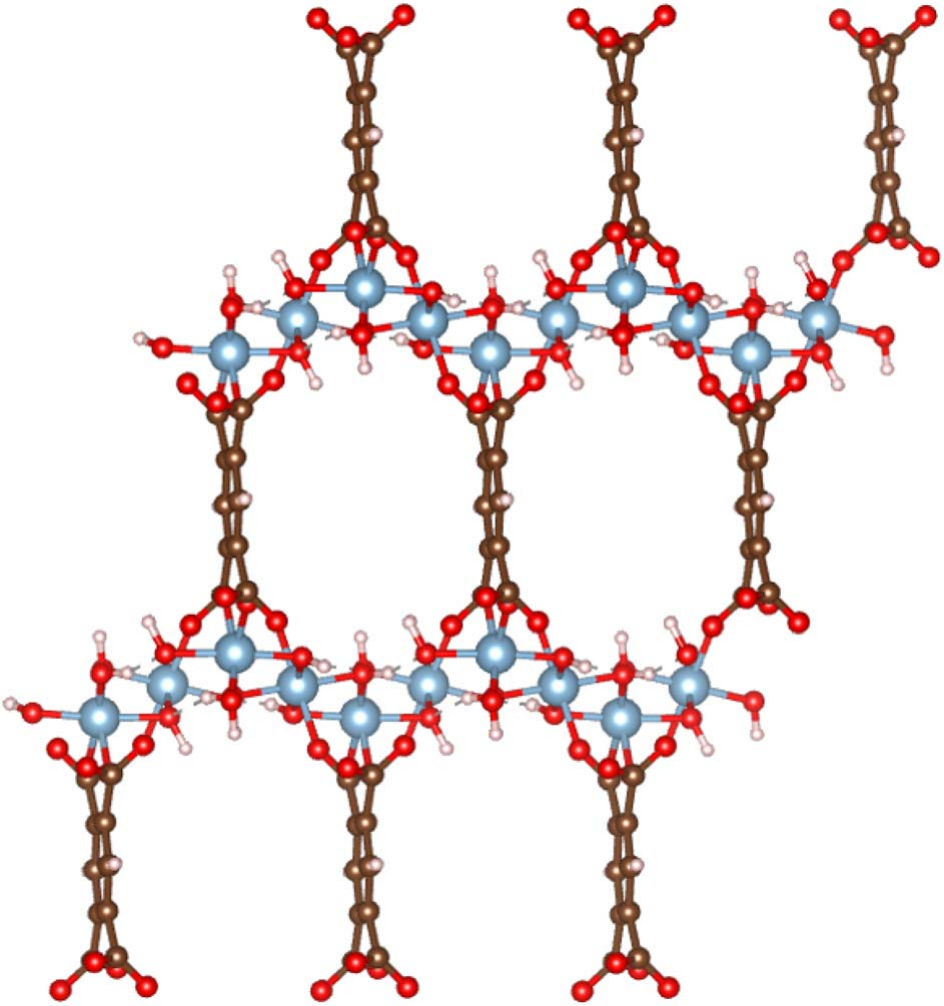
The atomistic structure of MOF MIL-120, where the red balls represent O atoms, blue ones represent Al atoms, white ones represent H atoms, and brown ones represent C atoms.

MIL-120, a robust microporous aluminum 1,2,4,5-benzene tetracarboxylate framework, has recently been reported by Chen *et al.*^22^ as a CO_2_ capture material candidate for its relatively moderate isosteric enthalpy of adsorption (≈−40 kJ mol^−1^), good CO_2_/N_2_ selectivity, and long-term stability under real conditions (see Fig. 1). Classical force field simulations of gas adsorption in Al-based MOFs often show significant discrepancies from experimental measurements,^23–25^ necessitating the use of empirical scaling factors. MIL-120 is no exception in this regard. For instance, Chen *et al.*^22^ set the energy parameters of Al to zero in their classical GCMC simulations to fit the experimental data. Naturally, simulations of MIL-120 are particularly challenging due to the structural complexity of the framework, considering the high density of µ_2_-OH groups, as well as the accessible aromatic rings decorating the channel surface, which play an essential role in the CO_2_ interaction with the atoms of the MOF. This work was inspired by the recent work of Li *et al.*,^26^ who developed an empirical force field to correct the UFF force field for Al-containing MOF. This force field showed a remarkable improvement for all Al-containing MOFs for which experimental isotherms were available, except for MIL-120. For this MOF, Li *et al.*^26^ could not capture the adsorption of CO_2_ correctly. Therefore, this represents an interesting MOF to study using a machine learning force field.

In this work, we present a general computational workflow, using CO_2_ adsorption in MIL-120 as a case study, to establish a machine-learning potential for predicting adsorption isotherms using grand-canonical Monte Carlo simulations. Our workflow assumes a flexible MOF and aims to predict CO_2_ adsorption isotherms with DFT-level accuracy. The final training set contains only around 3000 DFT single-point calculations, with training errors below 0.1 meV per atom for energies and below 20 meV Å^−1^ for intermolecular forces. The results illustrate the importance of dynamic changes in the chemical environment at the MOF pore surface on the gas adsorption behavior in MIL-120.

## Results and discussion

### Dynamic hydrogen-bond network

**Fig. 2:**
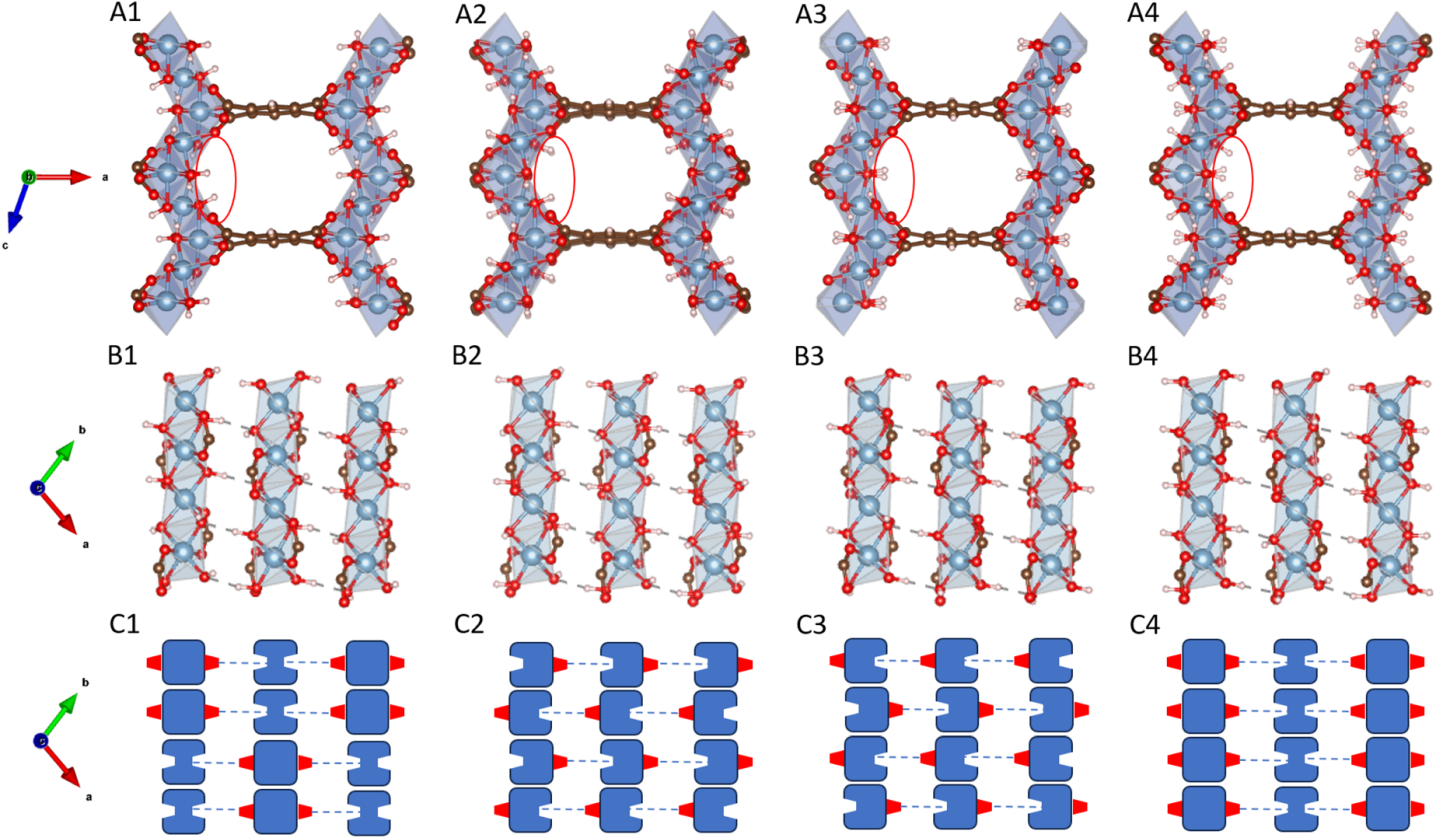
Different possible configurations of MIL-120, where the red balls represent O atoms, blue ones represent Al atoms, white ones represent H atoms, and brown ones represent C atoms: (A) distinct configurations have different types of OH adsorption sites on the pore surface; (B) the hydrogen-bonding networks between Al rod chains determine which µ_2_-OH groups are oriented toward the center of the pore. Each µ_2_-OH group on a rod chain forms a hydrogen bond with a neighboring chain's µ_2_-OH, where one serves as the donor and the other as the acceptor; (C) the schematic diagram of the hydrogen-bonding network between Al rods, where the red protruding blocks represent hydrogen bond donors and the grooves represent hydrogen bond acceptors. The hydrogen-bonding network is classified and named based on the hydrogen bond types of the central rod in the schematic diagram: str1 is AAAADDDD, str2 is ADDAADDA, str3 is DAADDAAD, and str4 is AAAAAAAA.

To understand the adsorption behavior of CO_2_ in MIL-120, we first need to analyze its internal hydrogen-bonding network. Chen *et al.*^22^ showed that MIL-120 has two stable structures. These structures differ in the orientation of the µ_2_-(OH) groups distributed along the infinitely extended Al–µ_2_-(OH)_2_ rod chains. The different orientations result in the four different structures shown in Fig. 2.

Specifically, hydrogen-bond networks are established between adjacent MOF rod chains through the orientations of these µ_2_-OH groups. Each µ_2_-OH group on a rod chain forms a hydrogen bond with a µ_2_-OH group on a neighboring chain, where one acts as a donor (D) and the other as an acceptor (A). The µ_2_-OH groups functioning as acceptors typically possess a free OH bond pointing into the MIL-120 pore, creating potential CO_2_ adsorption sites.

Once the orientation type of the µ_2_-OH groups on a single rod chain is determined, the orientations on adjacent rod chains can be inferred accordingly, enabling the construction of the complete hydrogen-bonding network across the MIL-120 unit cell.

However, due to the limitations of X-ray diffraction (XRD) experiments in accurately resolving hydrogen atom positions, the orientation of OH bonds in the µ-(OH) groups of MIL-120 cannot be directly determined. This uncertainty gives rise to multiple possible structural configurations. For example, in the work by Chen *et al.*,^22^ the OH orientation sequence in the rod chain repeat unit for the str1 structure is AAAADDDD, whereas for str2 it is ADDAADDA. In fact, beyond these two configurations, many other configurations are possible. For instance, as illustrated below, str3 exhibits DAADDAAD OH sequence, while str4 adopts a uniform AAAAAAAA pattern.

Performing CO_2_ adsorption energy calculations for each of these hydrogen-bonding variants, or treating them as rigid frameworks for GCMC simulations to obtain adsorption isotherms, would be computationally inefficient and practically infeasible. Therefore, it is necessary to develop more efficient strategies to address the configurational diversity arising from structural uncertainty in a reasonable manner.

### Training set construction strategy

To capture a sufficient and representative set of key MIL-120 configurations, while also encompassing the adsorption configurations of CO_2_ molecules, we designed and implemented a multi-faceted sampling strategy, which includes the following simulations:

(1) *Ab initio* molecular dynamics simulations of the MIL-120 empty framework to sample stable dynamic configurations of the framework structure (optional);

(2) Long-time molecular dynamics simulations of the MIL-120 empty framework based on the pre-trained MACE potential at different temperatures, to collect diverse dynamic framework configurations (MACE–MD);

(3) Grand Canonical Monte Carlo simulations of CO_2_ adsorption with a fixed framework using the UFF potential, to sample configurations of CO_2_ adsorption sites and configurations;

(4) Long-time molecular dynamics simulations of the MIL-120 with CO_2_ based on the pre-trained MACE potential at different temperatures, to collect diverse dynamic adsorption configurations.

#### Training the empty MIL-120

The first step is to create an initial training set for the empty MOF that optimally covers the configuration that can be expected from an MD simulation of the empty framework using our new force field. To estimate these configurations, we employ both AIMD and MACE–MD simulations to explore the configuration space. We use Smooth Overlap of Atomic Positions (SOAP) descriptors^27^ to quantify the similarity between these configurations.

Changes in the unit cell shape are one manifestation of MOF flexibility. In the present work, however, for MIL-120, no breathing behavior has been observed experimentally, and the unit cell does not undergo significant deformation under ambient conditions. Therefore, we only used NVT simulations in both AIMD and MACE–MD. For MOFs that do show changes in the unit cell shape, sampling using the Rahman–Parrinello MD scheme can be straightforwardly incorporated into our workflow by including stress in the MACE training parameters, without altering the overall procedure presented here.

**Fig. 3:**
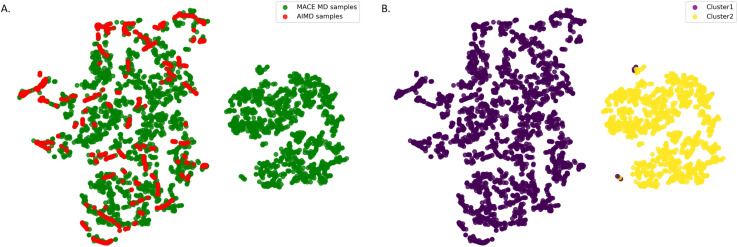
Two-dimensional projection of the SOAP structural descriptor space for empty MIL-120 configurations: (A) the green dots represent configurations that MACE–MD generates at 300 K and the red dots those generated by AIMD. The figure shows that AIMD and MACE–MD do not sample the same parts of the configuration space; (B) unsupervised learning shows that the configuration space has two distinct parts represented by yellow and purple dots.

Fig. 3 demonstrates the two-dimensional projection of the SOAP structural descriptor space for empty MIL-120 configurations. We used the t-distributed stochastic neighbor embedding (t-SNE) method, which preserves local similarities within the data, ensuring that similar structures are mapped close to each other in the 2D space. The plot clearly reveals that the configurational space is divided into two distinct regions, indicating a significant difference between the configurations sampled by AIMD simulations and those from simulations based on the pre-trained MACE potential. This highlights a key limitation of AIMD-based sampling. Due to its high computational cost, simulations are typically limited to short time scales, making it difficult to capture the full range of structural evolution and configurational diversity of the MOF framework starting from a single initial structure at finite temperature. From this, we selected the 800 most distant (and thus maximally diverse) points as the initial training set for the empty framework.

#### Training of MIL-120 with CO_2_

When analyzing the MOF configurations with CO_2_ adsorbed, we employed the SOAP descriptor not only to capture the structural diversity of the MOF frameworks but also to focus on the adsorption sites and configurations of CO_2_ molecules. The saturation loading is 12 CO_2_ per cell. We classified them into four categories based on the number of adsorbed CO_2_ molecules: 1, 4, 8, and 12. This classification was designed to simulate the complete adsorption isotherm comprehensively. For each loading, we selected the most diverse 200 configurations into the training sets.

### Active learning workflow

Although the initial sampling phase aims to capture a diverse set of configurations on the potential energy surface, fine-tuning a robust and highly accurate machine learning potential through a single round of sampling and training is impractical. However, many MLP studies on MOFs have not employed active learning to complement the missing information across the entire potential energy surface.^13,15,18,28^ Such information is essential for accurately evaluating the heat of adsorption and further simulating adsorption isotherms. We noticed that Sharma and Sanvito^29^ defined structural descriptors based on bond lengths, bond angles, and dihedral angles to screen diversity during the active learning process for MOF structures. However, such a classification depends on the specific MOFs, requiring different definitions of bond lengths, angles, and dihedrals for different structures, which is not suitable for a large-scale MOF structures training workflow. To address this limitation, we implement a two-step active learning strategy that combines force deviation and diversity analysis to explore previously unvisited regions of configuration space, thereby progressively enhancing the accuracy and transferability of the potential.

As a first screening criterion, we employed the variance in atomic forces predicted by an ensemble of machine learning potentials to identify regions of high uncertainty. In each training iteration, we generate four sets of machine learning potentials from the same training set but with different random seeds. The interatomic force deviation across multiple random seed models can be expressed as follows:1
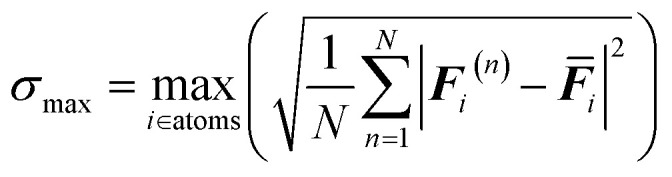
where ***F***_*i*_^(*n*)^ represents the force prediction on atom *i* by MACE model *n*, ***F̄***_*i*_ represents the average forces over all the MACE potential models, and the deviation serves as a criterion for structural selection. Those structures with force deviations between 0.06 and 0.24 eV Å^−1^ were selected as candidate configurations, depending on the users' desired level of accuracy for the final target MLP. Candidate configurations were then evaluated using SOAP descriptors to quantify structural diversity, and representative structures were selectively added to the training dataset.

Our results give a significantly better accuracy with an order of magnitude less training data compared to previous results.^12,21^ These studies also employ active learning, but they differ in the way configurations are selected for which additional DFT labeling is performed. Typically, these candidate structures were randomly sampled from the set of configurations that have too high errors in the forces or selected as the final frames of short MD simulations starting from a large number of randomly perturbed initial structures. We employ a different approach to avoid random sampling. We use a SOAP structural descriptor to quantify the similarity of the set of inaccurate configurations. We then perform our DFT labeling for a set of 200 most diverse configurations for each system that optimally span this set.

**Fig. 4:**
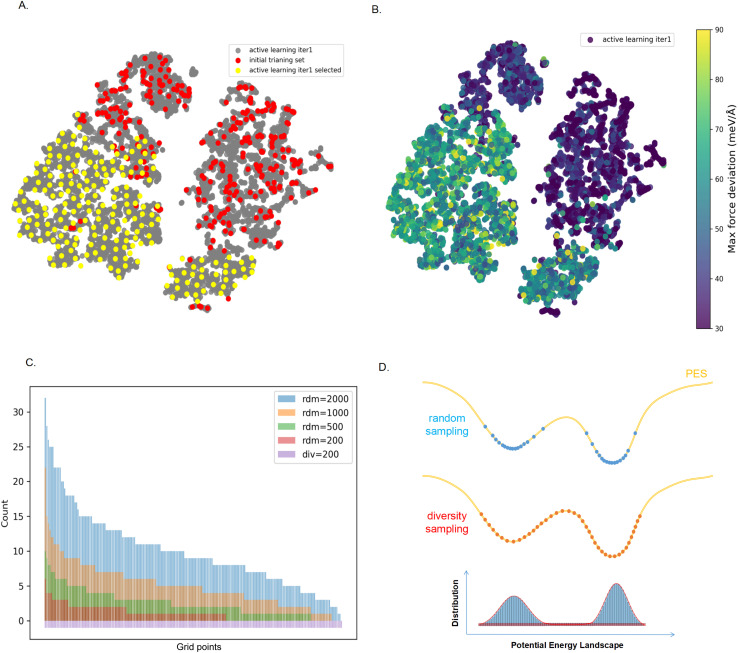
The sampling configuration space explored in the first iteration of active learning. (A) The gray dots represent all the sampled configurations obtained in the first iteration of active learning for MOF with one CO_2_ molecule. The red dots represent configurations from the initial training set, and the yellow dots represent the 200 most diverse configurations, which are added to the training set. (B) The sampling configuration space explored in the first iteration of active learning, color-coded by the estimated error of the prediction of the forces (maximum force deviation). (C) Diversity of the 200, 500, 1000, and 2000 randomly selected structures among those for which the error of the force field is too large. Regions with large force deviations were divided into 200 grid points, and 200 structures were selected to be uniformly distributed across these points using a diversity-based selection method. For a 2000 randomly selected configuration, we have at least one configuration in 197 grid points. (D) Illustration of the difference in random sampling and most diverse sampling for an MD simulation. The figure shows the sampled structures on the potential energy surface. Consequently, random sampling from the trajectory requires many more structures to cover the potential energy surface compared to uniform sampling based on diversity.

Fig. 4 shows the sampling of our MOF loaded with one CO_2_ molecule during the first round of active learning. The red dots in Fig. 4A are the first set of configurations for which we fine-tuned the Mace force field, and the grey dots are the configurations from a long MD trajectory we generated from the first round of fine-tuning. Fig. 4B is the same figure but now color-coded with the estimated accuracy of this force field. In our first round of active learning, we selected the set of configurations with poor accuracy. We determined the set of configurations that optimally cover this set, which are the yellow dots in Fig. 4A. In Fig. 4C, we compare our sampling with a random sampling from the same set of configurations, *i.e.*, how many random samples do we need to select to cover the same diverse configuration space? These results show that even with 2000 configurations, we do not cover the entire configuration space.

Fig. 4D illustrates the reason for this. The yellow line is a sketch of the energy landscape of a part of our MD trajectory. If we carry out a random sampling, we will mainly select the low-energy configurations. For an accurate MD trajectory, not only do the low-energy configurations matter, but the barriers are equally important. Our diversity algorithm ensures that representative configurations of these barriers are included in the training set. However, with random sampling, the probability of selecting such a high-energy configuration is (exponentially) small, and at the same time, it selects many configurations that add very little new information to our active learning. Hence, one needs an order of magnitude more configurations to have such a conformation selected during any of the learning cycles. In our example, even with 10 times more samples, we did not sample all relevant barriers. This is the main reason why we can get significantly better accuracy with far fewer DFT single-point calculations.

These results also highlight that AIMD simulations with only 10 000 DFT steps explore only a small fraction of the conformational space of such complex, dynamic structures. As shown in Fig. 4A, we can even omit all AIMD conformations from the training set, because sampling based on the pretrained MACE model already covers the conformational space explored by AIMD. For the hydrogen-bond network in MIL-120, fewer than 3000 single-point DFT calculations are sufficient to achieve an accurate description of the system. This approach offers substantial savings in computational resources compared to most MLP training strategies that rely heavily on conformations extracted directly from AIMD trajectories.

Fig. 6 illustrates the distribution of the training set within the entire sampling space. This space includes not only all structural frames obtained during the initial data sampling phase for training but also all frames extracted from long-time molecular dynamics and GCMC simulation trajectories generated using the trained models. By comparing these distributions, one can evaluate the extent to which the training set covers the overall configurational space and assess the model's generalization capability in realistic simulation tasks. These simulation tasks includes not only simple single-energy calculations and NVT MD simulations but also hybrid MD–GCMC simulations.

### Validation MLP with DFT results

**Fig. 5:**
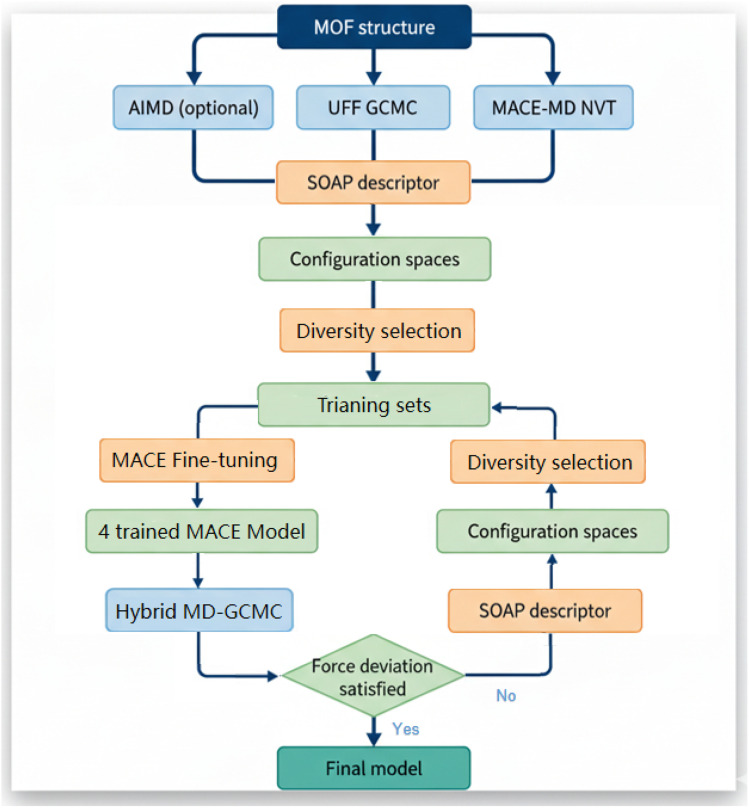
The workflow of diversity sampling based MACE machine learning potential fine-tuning method. For the system studied in this work, we selected 800 structures for the initial training set of the empty framework. For structures with CO_2_ adsorption, 200 configurations were added for each loading. During each round of active learning, 200 configurations at different loadings were selected and added until the MLP converged.

Our whole MLP training workflow is shown in Fig. 5. After obtaining the final converged MLP, we implemented an additional validation dataset by extracting configurations from the extra-long MACE–MD simulation trajectory for 1500 ps in different loadings. From these simulations, we extracted 200 randomly selected structures from MOF with 0 CO_2_, 1 CO_2_, and 4 CO_2_ molecules for additional DFT labeling to compare with MACE-evaluated energies and forces.

**Fig. 6:**
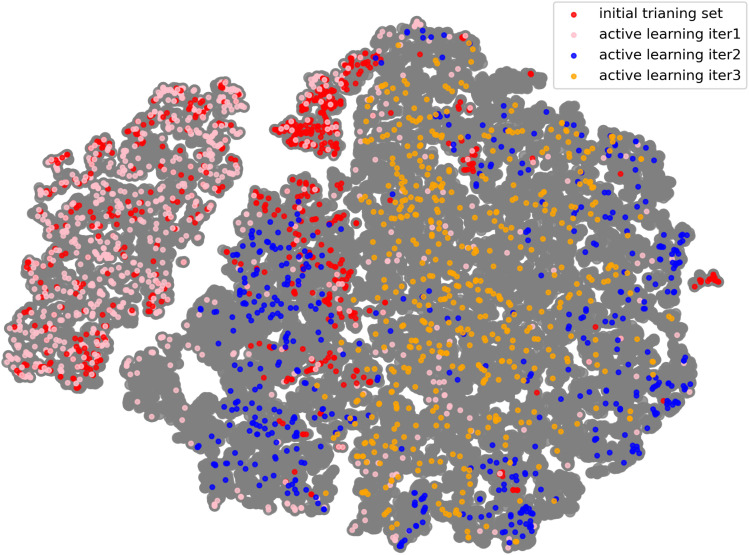
The distribution of the training set within the entire sampling space. The gray dots represent all the sampled configurations obtained in this work. The red dots represent configurations from the initial training set, and through the iterative active learning procedure, three additional training sets have been added (pink, blue, and yellow, respectively). For the frames with CO_2_, we only analyzed the structural descriptors of the MOF framework.

**Fig. 7:**
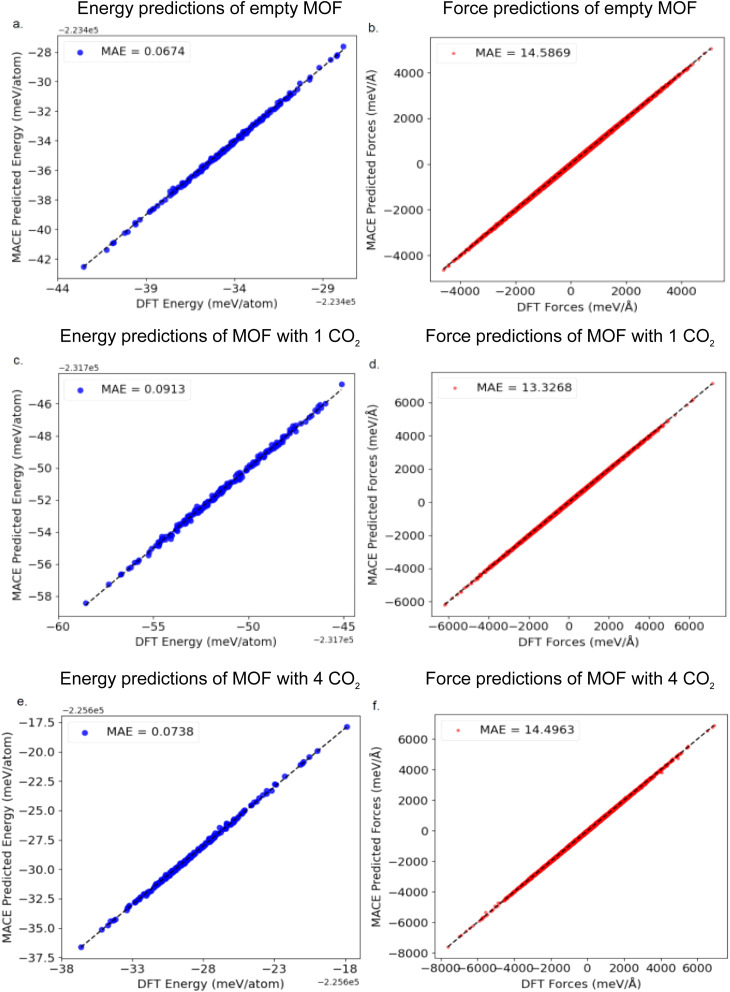
Comparison between the fine-tuned MACE potential and DFT calculations in terms of energy and interatomic force predictions. (a) and (b) For the empty MOF framework system, the prediction errors MAE of the MACE model for energy and atomic forces are 0.067 meV per atom and 14.58 meV Å^−1^, respectively. (c) and (d) In the system with one adsorbed CO_2_ molecule, the corresponding errors for energy and forces are 0.091 meV per atom and 13.32 meV Å^−1^, respectively. (e) and (f) In the system with four adsorbed CO_2_ molecule, the corresponding errors for energy and forces are 0.074 meV per atom and 14.50 meV Å^−1^, respectively.

Fig. 7 presents a comparison between the fine-tuned MACE potential and DFT calculations in terms of energy and interatomic force predictions, demonstrating excellent agreement. For the empty MOF framework system, the prediction errors MAE of the fine-tuned MACE model for energy and atomic forces are 0.07 meV per atom and 14.58 meV Å^−1^, respectively. In the system with one adsorbed CO_2_ molecule, the corresponding errors for energy and forces are 0.07 meV per atom and 13.32 meV Å^−1^, respectively. Combined with the previously shown broad and well-distributed coverage of the configurational space, this further confirms that the constructed MACE potential effectively captures the dynamic structural variations of the MIL-120 framework. These error bars are an order of magnitude smaller than typically reported for MLP describing MOFs.^12,13,15,18^ Therefore, this model serves as an efficient and reliable alternative, enabling feasible simulations of experimentally observable macroscopic properties, such as adsorption isotherms and heats of adsorption, while maintaining accuracy close to the DFT level.

### CO_2_ adsorption dynamics

#### Hydrogen-bond network

One of the core objectives in fine tuning the MACE potential is to accurately capture the dynamically evolving hydrogen-bonding network within the MIL-120 framework—an essential prerequisite for precise modeling of CO_2_ adsorption behavior. To this end, we performed NVT MACE–MD simulations for 1500 ps at multiple temperatures using our trained MACE potential, starting from the str1 configuration of MIL-120. During these simulations, we tracked the configurational evolution of each µ_2_-OH bond in the system.

**Fig. 8:**
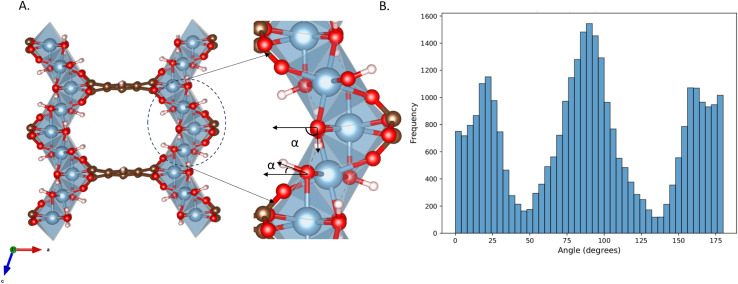
(A) The figure shows the definition of *α*, which is defined as the angle between the projection vector of the OH bond onto the *xz*-plane and the *x*-axis; (B) observed distribution of *α* as obtained from a MACE–MD simulation of the empty MIL-120 structure at 303 K.

To quantitatively characterize the directionality of each OH bond, we computed the angle *α* between the projection vector of the OH bond onto the *xz*-plane and the *x*-axis. As shown in the Fig. 8, we obtained the statistical distribution of these angles over a 0.1 ns timescale.

The angle range was divided into three intervals: 0° to 45°, 45° to 135°, and 135° to 180°. OH bonds falling within the first and third intervals were defined as “free OH” pointing toward the pore center, functioning as hydrogen bond acceptors. OH bonds within the middle interval were oriented toward neighboring rod chains, serving as hydrogen bond donors. The distribution of OH orientations among the three categories remained approximately in a 1 : 2 : 1 ratio. This observation is consistent with our earlier structural analysis, wherein two adjacent µ_2_-(OH) groups form a stable hydrogen bond, while the remaining OH bond points into the pore, acting as a potential CO_2_ adsorption site.

**Fig. 9:**
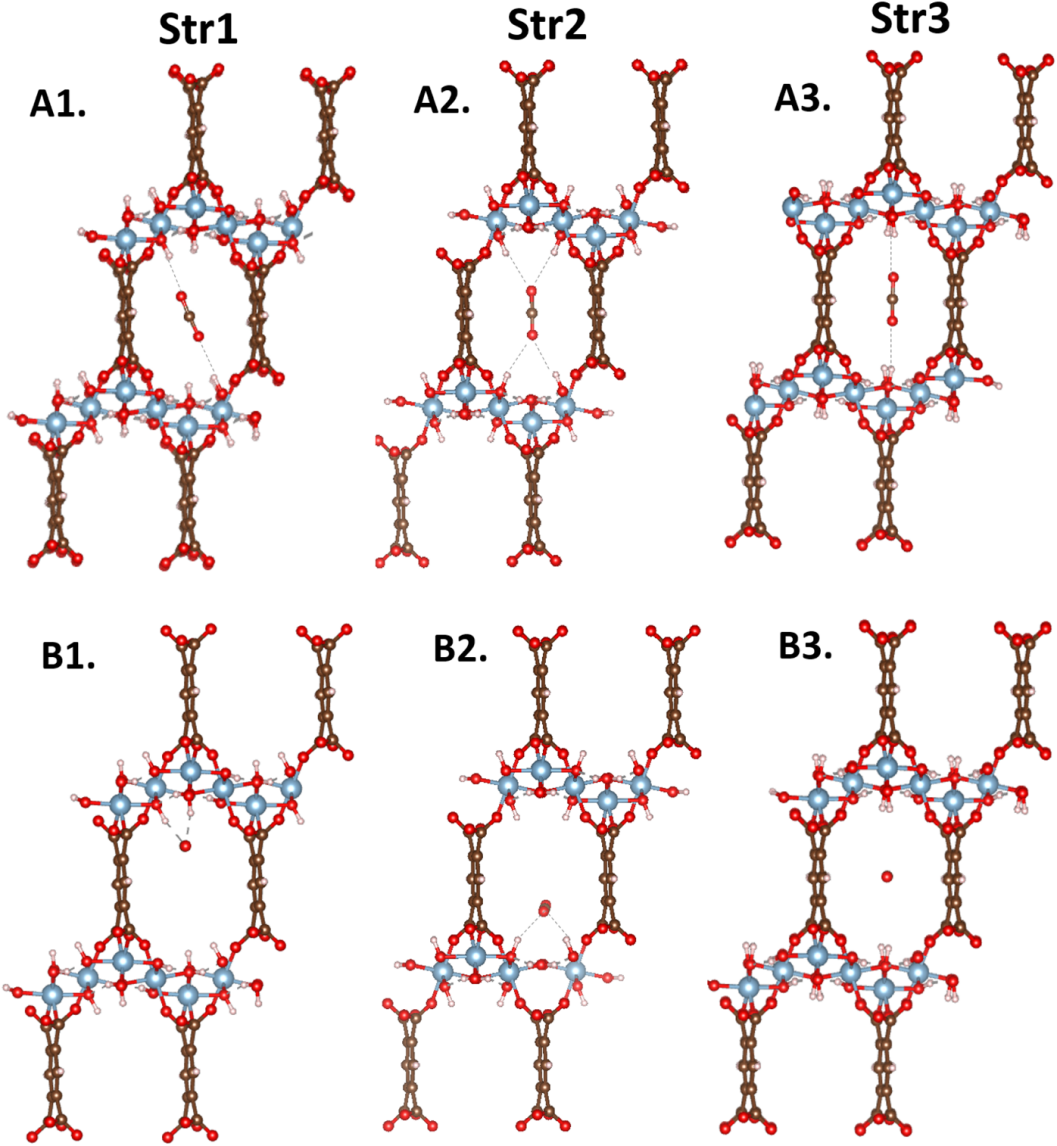
In different local structures of MIL-120, the adsorption sites of CO_2_ are primarily classified into three representative structural types based on the spatial arrangement of OH groups on the pore surface. Within each type, CO_2_ molecules exhibit two typical adsorption configurations: parallel (A) and vertical (B) relative to the channel direction.

In MIL-120, the number of OH groups pointing toward the pore center is constant. Changes in their spatial distribution within the pore have a large impact on the interactions with CO_2_ molecules. Certain adsorption sites allow a single CO_2_ molecule to interact simultaneously with multiple OH groups, thereby significantly affecting its adsorption capacity. Fig. 9 shows the CO_2_ adsorption sites and Table 1 the corresponding DFT-calculated binding energies for the three main structural units (str1, str2, and str3 in Fig. 8) in MIL-120. It is evident that the introduction of CO_2_ induces a configurational response on the pore surface, altering the local morphology of the pore environment.

**Table 1:** DFT binding energies for different types of structures

Structures	Binding energy (kJ mol^−1^)
Str1_parallel	−41.07
Str1_vertical	−36.17
Str2_parallel	−46.95
Str2_vertical	−44.56
Str3_parallel	−34.77
Str3_vertical	−33.56

**Fig. 10:**
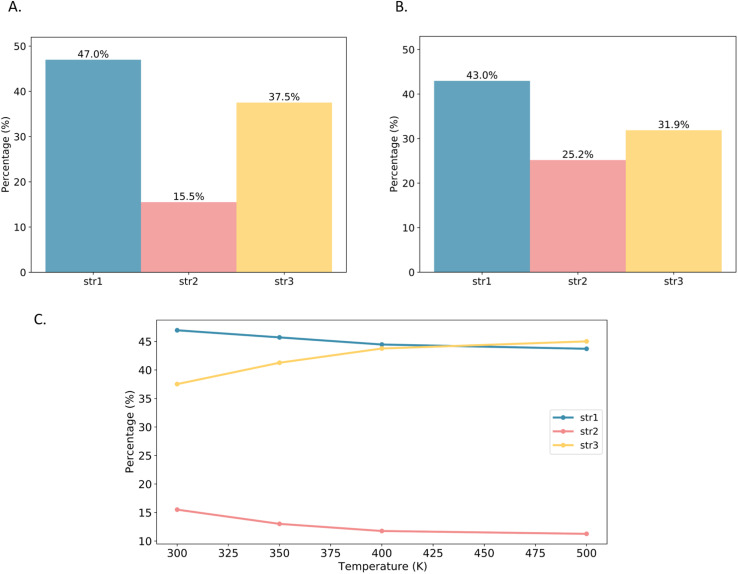
(A) The structure ratio of empty MIL-120 at 300 K; (B) the structure ratio of MIL-120 adsorbed with one CO_2_ molecule at 300 K; (C) the structure ratio of empty MIL-120 at different temperatures.

Based on this analysis, the MIL-120 framework was divided into these three structural units. Molecular dynamics simulations of CO_2_-adsorbed MOF structures at various temperatures were conducted to statistically track changes in the proportions of each structural unit. As shown in the Fig. 10, str1 dominates in the empty MIL-120 framework; however, upon CO_2_ adsorption, the proportion of str3 gradually decreases while that of str2 notably increases. This shift is primarily attributed to the interactions between CO_2_ molecules and the OH groups on the pore surface, which perturb the equilibrium of the dynamic hydrogen-bond network. Due to the variation in CO_2_ binding energies across different structures, the adsorption process tends to induce a structural transition favoring the formation of str1 and str2, which offer stronger adsorption sites. To investigate the effect of temperature on the intrinsic framework behavior, we performed angle-based structural analysis on the empty MIL-120 framework at various temperatures. The results show that as the temperature increases, the proportion of str1 gradually decreases, str2 declines rapidly, while str3 increases significantly. This indicates a temperature-driven reorganization of the hydrogen-bond network toward more disordered structures. This phenomenon is a key reason why traditional rigid-framework models fail to accurately simulate CO_2_ adsorption in MIL-120, highlighting the critical importance of accurately modeling structural flexibility and the dynamic hydrogen-bond network for reliable adsorption performance descriptions.

By performing molecular dynamics simulations on both the empty MOF framework and the MOF structure with adsorbed CO_2_ molecules, we calculated the heat of adsorption of CO_2_ in MIL-120 using the following formula:

2 Δ*H*_ads_ = 〈*E*_MOF+CO_2__〉 − 〈*E*_MOF_〉 − 〈*E*_CO_2__〉 − *RT*

where 〈*E*_MOF+CO_2__〉 is the ensemble-averaged total energy of the MIL-120 structure with one adsorbed CO_2_ molecule, 〈*E*_MOF_〉 is the ensemble-averaged energy of the empty MIL-120 framework, and 〈*E*_CO_2__〉 is the ensemble-averaged energy of a single CO_2_ molecule in the gas phase.

The calculated heat of adsorption is −41.9 kJ mol^−1^, which is in excellent agreement with experimental value (−40.2 kJ mol^−1^). If we compare this with the value obtained by UFF (−52.3 kJ mol^−1^) or the modified UFF (−50.2 kJ mol^−1^), we see that this represents a significant improvement.

To more accurately capture the dynamic structural response of the MOF framework during gas adsorption, we incorporated a molecular dynamics-coupled strategy into the GCMC simulations. Specifically, prior to each CO_2_ insertion or deletion attempt, a short molecular dynamics simulation was performed on the entire system. This approach updates the framework configuration, allowing it to more realistically reflect the flexibility changes that occur during the adsorption process.

**Fig. 11:**
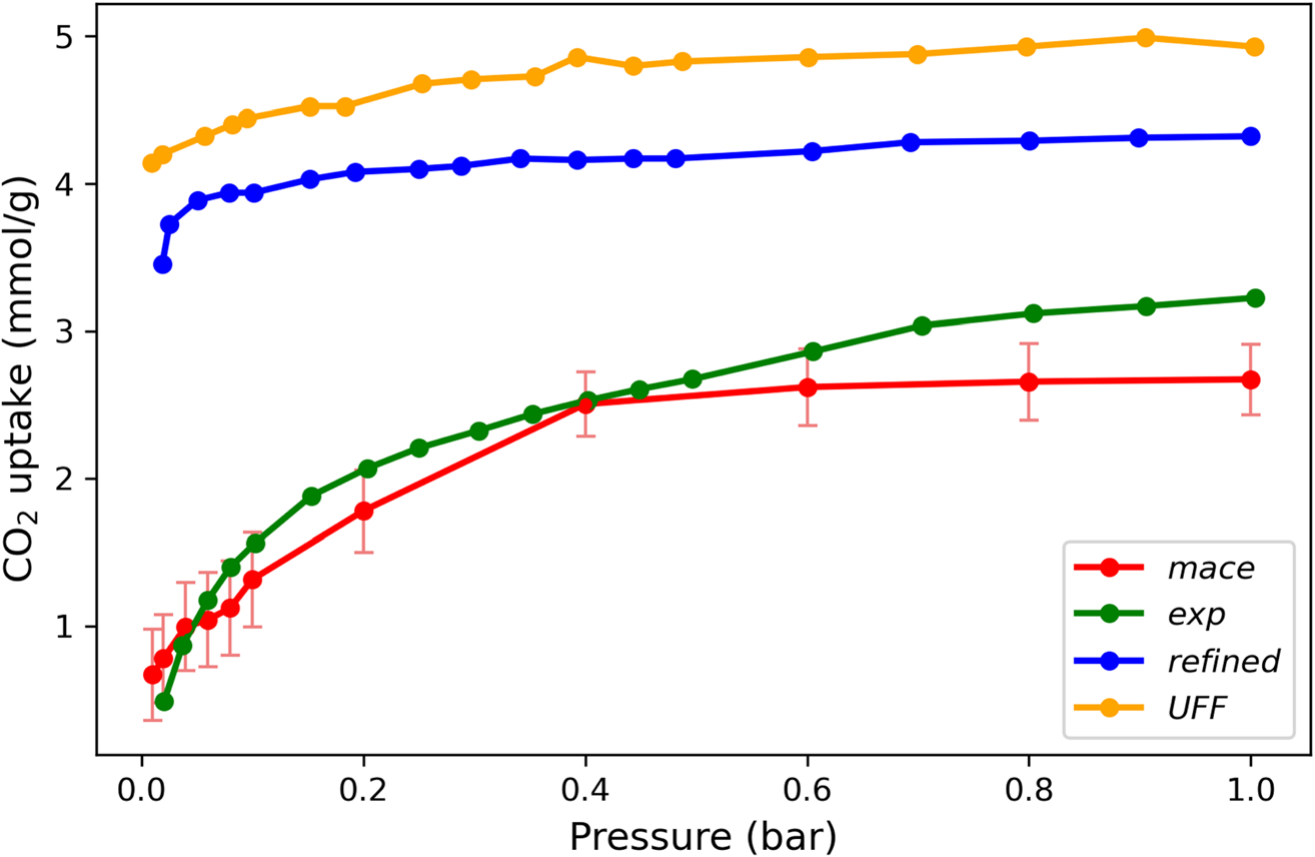
Comparison of the simulated and experimental CO_2_ adsorption isotherms at 303 K. The green curve represents the experimental CO_2_ adsorption isotherm. The red curve shows the result from this work using GCMC simulations with a fine-tuned MACE potential on a flexible framework. The yellow curve corresponds to GCMC simulations with a fixed framework using the UFF force field, while the blue curve shows results from a fixed-framework simulation using a refined force field for aluminum centers.^26^

Fig. 11 compares the predicted CO_2_ adsorption isotherms in MIL-120 at 303 K using different simulation methods. The results show that the MD–GCMC approach, which accounts for framework dynamics, provides adsorption predictions that align more closely with experimental data. This highlights the significant role of structural flexibility in governing the adsorption performance of this material.

It is instructive to compare our results with the simulation results of Chen *et al.*^22^ It is well known that the UFF force field overestimates the results for Al-containing MOFs. For example, Boyd *et al.*^6^ used a scaling parameter to reduce the van der Waals interactions. Chen *et al.*^22^ could obtain excellent agreement with the experimental isotherms of CO_2_ in MIL-120 by setting the van der Waals interaction of CO_2_ with the Al atoms to zero. Li *et al.*,^26^ however, argued that it is not the Al atoms that have the incorrect parameters in UFF, but the O and C atoms next to Al. However, all these calculations assume that the MOF is rigid. This work shows that for MIL-120, the explanation is much more subtle. It is not only a matter of changing the UFF parameters, but it is intrinsically related to the dynamics of the hydrogen bond network that cannot be captured if we assume a MOF is rigid.

## Concluding remarks

The reason why previous studies failed to describe the adsorption behavior in MIL-120 correctly is quite remarkable. The reported crystal structure has an ideal binding site in which all four hydrogens are optimally positioned to form hydrogen bonds with both oxygens of the CO_2_ molecule. Our *ab initio* simulations showed that at ambient conditions, thermal disorder makes it unlikely that, at room temperature, such optimal ordering persists. What makes MIL-120 special is that the size of the cavity and the positions and orientations of the hydrogens create a unique and very strong binding site in which four hydrogens can interact with the two oxygens of CO_2_. We do expect that if the experiments were carried out at much lower temperatures, the agreement with the predictions of these classical force fields would be much better.

Also, it is important to realize that the effect of the hydrogen is amplified because four hydrogens collectively interact with CO_2_, in most MOFs, it will only be one. In such a case, movements of the hydrogen will have little, if any, impact on the adsorption behavior. Therefore, we do not expect similar failures to describe the CO_2_ adsorption in most other MOFs in classical force fields with a rigid framework.

In this work, we developed a comprehensive and efficient computational workflow to fine-tune an MACE potential for modeling gas adsorption in flexible MOFs, which, in the case of MIL-120, can accurately capture the dynamic hydrogen-bond network critical to CO_2_ adsorption. By integrating multiple sampling strategies, we constructed a diverse and representative training dataset, further refined through an active learning loop. This active learning process aimed to ensure that we carry out an optimal number of DFT calculations, where we define optimal as a compromise between available CPU time, accuracy, and the diversity of configurations that may need to be sampled. In this case, we carried out about 3000 single-point DFT calculations.

For carbon capture applications, CO_2_ is an important component. An equally important component in carbon capture applications in H_2_O. Our results for MIL-120 show that the MACE potential reliably captures that CO_2_ adsorption induces a reorganization of the hydrogen-bonding network, altering the pore environment in ways that rigid-framework models cannot capture. These observations are fascinating, but unique to MIL-120. However, for the adsorption of H_2_O, we expect this to be the norm. An optimal orientation of a single hydrogen of the MOF can facilitate the formation of a hydrogen-bond network for H_2_O.

The configuration number of the training set is two orders of magnitude larger than that used by Lim *et al.*^18^ to describe the adsorption of CO_2_ in MOFs with open metal sites. There are a few reasons why these numbers are so different. First, we computed the complete isotherm, while Lim *et al.*^18^ focused on a single CO_2_ molecule. A more important difference is that Lim *et al.*^18^ assumed that the MOF was rigid and focused on fine-tuning the MACE potential at the binding site. This is a very efficient strategy for those MOFs for which the binding site is known, but it would not work in the case of MIL-120. This study highlights the necessity of accounting for framework flexibility and dynamic chemical environments in realistic MOF adsorption simulations. The proposed machine learning-based workflow offers a transferable strategy for studying other flexible MOF systems and provides new insights for the design of MOF materials for direct air capture and related applications.

All our DFT results can be found on Zenodo. If more studies make their DFT calculations available, these force field models can be systematically improved for these metal–organic frameworks. This will eventually reduce the number of DFT calculations needed to fine-tune these models.

## Methodology

### Force field sampling

The initial training set of MOFs includes the pristine MOF and the MOF with CO_2_ configurations generated from MD trajectories. Simulations were performed using the pretrained MACE force field MACE-MP-0b2 (ref. 30) calculator in the canonical ensemble with the LAMMPS package,^31^ employing a Nose–Hoover thermostat with a time step of 0.5 fs over a total duration of 50 ps. Trajectories were sampled at three temperatures: 100, 300, and 500 K to capture thermally accessible configurations across a wide range of thermal conditions. Temperatures above 500 K were avoided to minimize the likelihood of generating unphysical structures.

The initial training set of MOF with CO_2_ configurations was also generated from UFF GCMC simulations at subsequent pressure points. They were performed by the Isotherm work chain in the aiida-lsmo plugin^32^ with RASPA^33^ molecular simulation software. The optimized framework geometries were kept rigid in classical simulations. We considered van der Waals and electrostatic interactions to describe the energy surface, represented respectively by the Lennard-Jones (LJ) potential and Coulomb interactions. The density-derived electrostatic and chemical (DDEC) method^34^ is used to compute the partial charges on the atoms of the MOF frameworks. The Ewald summation technique was used to model the Coulomb interaction. The TraPPE force field^35^ was selected to model gas–gas interactions. The Lennard-Jones parameters were taken from the Universal Force Field (UFF)^36^ to model the interactions of CO_2_ with the framework atoms.

The hybrid MD-GCMC simulations were performed with the LAMMPS package. At a temperature of 300 K, employing a Nose–Hoover thermostat with a time step of 0.5 fs, the ideal gas reservoir is defined by specifying different pressures. Throughout the simulation, NVT MD simulations are performed, with one GCMC insertion/deletion attempt every 5 steps. The positions of CO_2_ molecules are updated in real time during the MD simulation; there are no translation or rotation Monte Carlo move attempts. The chemical potential, as defined in Lammps, is calculated by pressure according to the following equation:3
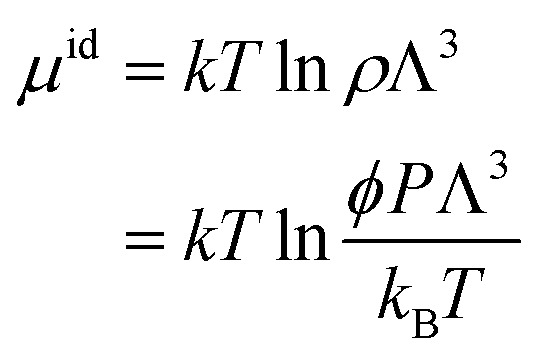
where *k*_B_ is the Boltzmann constant, *P* is the pressure, and *ϕ* is the fugacity coefficient. In our simulations, the gas pressures do not exceed 1 bar, so deviations from ideal gas behavior can be ignored. We set the fugacity equal to the pressure. The constant *Λ* is defined as the thermal de Broglie wavelength:4
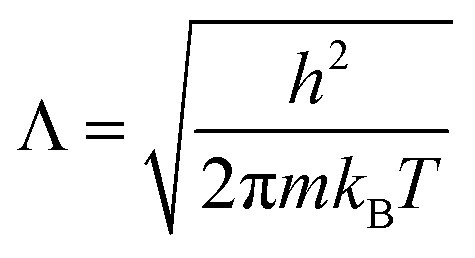
where *m* is the mass of CO_2_ molecule.

### DFT sampling and labeling

The quickstep code of the CP2K software (9.1 version)^37^ was used to perform DFT calculations for DDEC charge calculation, configuration sampling and labeling. These calculations were performed using Perdew–Burke–Ernzerhof (PBE) functional^38^ within the generalized gradient approximation, with Grimme's D3 corrections for van der Waals (vdw) interactions.^39^ The multigrid used for plane waves had a 4-level structure with a primary cutoff of 600 Ry, a relative cutoff of 50 Ry, and a progression factor of 3. We employed double-zeta DZVP-MOLOPT-SR contracted basis sets and GTH pseudopotentials to represent the electronic wave function. All the DFT simulations are sampled at the Gamma point.

*Ab initio* molecular dynamics (AIMD) sampling was performed in the canonical ensemble employing a Nose–Hoover thermostat at 300 K with a time step of 0.5 fs over a total duration of 5 ps.

### *K*-Means sampling and SOAP descriptor

To efficiently sample representative configurations from the molecular dynamics trajectories, atomic environments were first encoded using Smooth Overlap of Atomic Positions (SOAP) descriptors, computed with the Python package DScribe v2.1.1.^40^ A cutoff for the local region in angstroms is defined as *r*_cut_ = 6.0, and descriptor parameters *n*_max_ and *l*_max_ are set as 9 and 3, respectively. To construct training, validation, and test datasets, we applied *K*-means clustering to the SOAP feature space. The full configuration space was partitioned into *N* clusters, and the configuration closest to each cluster center was selected as a representative sample. This approach ensures that the final dataset spans the structural diversity observed in the simulation trajectories while minimizing redundancy. For visualization, the SOAP features were projected into two dimensions using the t-distributed Stochastic Neighbor Embedding (t-SNE) algorithm.

### MACE fine-tuning

Fine-tuning was performed on the pre-trained MACE-MP-0b2-medium model using the MACE package (version 0.3.13).^16,17^ The model architecture was retained from the original; it uses 128 channels with a maximum message-passing angular momentum quantum number of max_*L* = 1. Local atomic environments were defined using a cutoff radius of 5 Å, and interatomic distances were encoded using eight radial basis functions. Here, we assume that long-range electrostatic interaction can be ignored, even though our simulated systems include hydrogen bond networks, as demonstrated by Zhang *et al.*^41^ in their work on MLP for bulk water. This assumption yields good results. However, one should be cautious not to use our MLP in cases involving charge transfer, free ions, or external electric fields. In such cases, it is necessary to treat the charges separately. We applied the multihead replay fine-tuning mechanism to avoid catastrophic forgetting. A two-stage fine-tuning strategy was adopted to balance the learning of energy and force contributions. In the first stage, the loss weights were set to 1 : 100 for energy and force, respectively. In the second stage, the weights were adjusted to 10 : 1 to focus on accurate energy prediction. Optimization was performed with a batch size of 8 using an initial learning rate of 1 × 10^−3^ with a weight decay of 5 × 10^−7^. In the second stage, the learning rate was reduced to 1 × 10^−4^. Early stopping was employed with a patience parameter of 10 epochs to prevent overfitting.

## Author contributions

XJ and BS designed the project. XJ designed the training workflow. XJ and YL sampled and DFT labeled the training sets. KG and XZ performed data validation. The manuscript was written with contributions from all authors. All authors have approved the final version of the manuscript.

## Conflicts of interest

The authors declare no competing interests.

## Supplementary Material

SC-017-D5SC09058J-s001

## Data Availability

The training sets and fine-tuned MACE model can be found in detail on Zenodo https://zenodo.org/records/16903301. Supplementary information (SI) is available. See DOI: https://doi.org/10.1039/d5sc09058j.

## References

[cit1] Li J.-R., Sculley J., Zhou H.-C. (2012). Metal–organic frameworks for separations. Chem. Rev..

[cit2] Moghadam P. Z., Li A., Wiggin S. B., Tao A., Maloney A. G., Wood P. A., Ward S. C., Fairen-Jimenez D. (2017). Development of a Cambridge Structural Database subset: a collection of metal–organic frameworks for past, present, and future. Chem. Mater..

[cit3] Kumar A., Madden D. G., Lusi M., Chen K.-J., Daniels E. A., Curtin T., Perry IV J. J., Zaworotko M. J. (2015). Direct air capture of CO_2_ by physisorbent materials. Angew. Chem., Int. Ed..

[cit4] Boyd P. G., Lee Y. J., Smit B. (2017). Computational development of the nanoporous materials genome. Nat. Rev. Mater..

[cit5] Lee S., Kim B., Cho H., Lee H., Lee S. Y., Cho E. S., Kim J. (2021). Computational screening of trillions of metal–organic frameworks for high-performance methane storage. ACS Appl. Mater. Interfaces.

[cit6] Boyd P. G., Chidambaram A., García-Díez E., Ireland C. P., Daff T. D., Bounds R., Gładysiak A., Schouwink P., Moosavi S. M., Maroto-Valer M. M. (2019). others Data-driven design of metal–organic frameworks for wet flue gas CO_2_ capture. Nature.

[cit7] Yazaydın A. O., Snurr R. Q., Park T.-H., Koh K., Liu J., LeVan M. D., Benin A. I., Jakubczak P., Lanuza M., Galloway D. B. (2009). others Screening of metal- organic frameworks for carbon dioxide capture from flue gas using a combined experimental and modeling approach. J. Am. Chem. Soc..

[cit8] Dzubak A. L., Lin L.-C., Kim J., Swisher J. A., Poloni R., Maximoff S. N., Smit B., Gagliardi L. (2012). Ab initio carbon capture in open-site metal–organic frameworks. Nat. Chem..

[cit9] Haldoupis E., Borycz J., Shi H., Vogiatzis K. D., Bai P., Queen W. L., Gagliardi L., Siepmann J. I. (2015). Ab initio derived force fields for predicting CO_2_ adsorption and accessibility of metal sites in the metal–organic frameworks M-MOF-74 (M= Mn, Co, Ni, Cu). J. Phys. Chem. C.

[cit10] Zhang L., Han J., Wang H., Car R., E W. (2018). Deep potential molecular dynamics: a scalable model with the accuracy of quantum mechanics. Phys. Rev. Lett..

[cit11] Batzner S., Musaelian A., Sun L., Geiger M., Mailoa J. P., Kornbluth M., Molinari N., Smidt T. E., Kozinsky B. (2022). E (3)-equivariant graph neural networks for data-efficient and accurate interatomic potentials. Nat. Commun..

[cit12] Achar S. K., Wardzala J. J., Bernasconi L., Zhang L., Johnson J. K. (2022). Combined deep learning and classical potential approach for modeling diffusion in uio-66. J. Chem. Theory Comput..

[cit13] Zheng B., Oliveira F. L., Neumann Barros Ferreira R., Steiner M., Hamann H., Gu G. X., Luan B. (2023). Quantum informed machine-learning potentials for molecular dynamics simulations of CO_2_'s chemisorption and diffusion in Mg-MOF-74. ACS Nano.

[cit14] Goeminne R., Vanduyfhuys L., Van Speybroeck V., Verstraelen T. (2023). DFT-Quality adsorption simulations in metal–organic
frameworks enabled by machine learning Potentials. J. Chem. Theory Comput..

[cit15] Yue Y., Mohamed S. A., Loh N. D., Jiang J. (2024). Toward a generalizable machine-learned potential for metal–organic frameworks. ACS Nano.

[cit16] Batatia I., Kovacs D. P., Simm G. N. C., Ortner C., Csanyi G. (2022). MACE: Higher Order Equivariant Message Passing Neural Networks for Fast and Accurate Force Fields. Adv. Neural Inf. Process. Syst..

[cit17] Batatia I. , Batzner S., Kovács D. P., Musaelian A., Simm G. N. C., Drautz R., Ortner C., Kozinsky B. and Csányi G., The Design Space of E(3)-Equivariant Atom-Centered Interatomic Potentials, arXiv, 2022, preprint, arXiv:2205.06643, 10.48550/arXiv.2205.06643 PMC11769842 39877429

[cit18] Lim Y., Park H., Walsh A., Kim J. (2025). Accelerating CO2 direct air capture screening for metal-organic frameworks with a transferable machine learning force field. Matter.

[cit19] Li N., Pang J., Lang F., Bu X.-H. (2024). Flexible metal–organic frameworks: from local structural design to functional realization. Acc. Chem. Res..

[cit20] Férey G., Serre C. (2009). Large breathing effects in three-dimensional porous hybrid matter: facts, analyses, rules and consequences. Chem. Soc. Rev..

[cit21] Goeminne R., Van Speybroeck V. (2025). Ab initio predictions of adsorption in flexible metal–organic frameworks for water harvesting applications. J. Am. Chem. Soc..

[cit22] Chen B., Fan D., Pinto R. V., Dovgaliuk I., Nandi S., Chakraborty D., García-Moncada N., Vimont A., McMonagle C. J., Bordonhos M. (2024). others A Scalable Robust Microporous Al-MOF for Post-Combustion Carbon Capture. Adv. Sci..

[cit23] Vujić B., Lyubartsev A. P. (2016). Transferable force-field for modelling of CO_2_, N2, O2 and Ar in all silica and Na+ exchanged zeolites. Model. Simulat. Mater. Sci. Eng..

[cit24] Pérez-Pellitero J., Amrouche H., Siperstein F. R., Pirngruber G., Nieto-Draghi C., Chaplais G., Simon-Masseron A., Bazer-Bachi D., Peralta D., Bats N. (2010). Adsorption of CO_2_, CH4, and N2 on zeolitic imidazolate frameworks: experiments and simulations. Chem.–Eur. J..

[cit25] Lin L.-C., Lee K., Gagliardi L., Neaton J. B., Smit B. (2014). Force-field development from electronic structure calculations with periodic boundary conditions: applications to gaseous adsorption and transport in metal–organic frameworks. J. Chem. Theory Comput..

[cit26] Li Y., Jin X., Moubarak E., Smit B. (2024). A refined set of universal force field parameters for some metal nodes in metal–organic frameworks. J. Chem. Theor. Comput..

[cit27] Bartók A. P., Kondor R., Csányi G. (2013). On representing chemical environments. Phys. Rev. B: Condens. Matter Mater. Phys..

[cit28] Liu S., Dupuis R., Fan D., Benzaria S., Bonneau M., Bhatt P., Eddaoudi M., Maurin G. (2024). Machine learning potential for modelling H 2 adsorption/diffusion in MOFs with open metal sites. Chem. Sci..

[cit29] Sharma A., Sanvito S. (2024). Quantum-accurate machine learning potentials for metal-organic frameworks using temperature driven active learning. npj Comput. Mater..

[cit30] Batatia I. , *et al.*, A foundation model for atomistic materials chemistry, arXiv, 2023, preprint, arXiv:2401.00096, 10.48550/arXiv.2401.00096 41230846

[cit31] Gissinger J. R., Nikiforov I., Afshar Y., Waters B., ki Choi M., Karls D. S., Stukowski A., Im W., Heinz H., Kohlmeyer A., Tadmor E. B. (2024). Type Label Framework for Bonded Force Fields in LAMMPS. J. Phys. Chem. B.

[cit32] Team, L. , An AiiDA Workflows for the LSMO Laboratory at EPFL, 2023, https://github.com/lsmo-epfl/aiida-lsmo, accessed: 2023

[cit33] Dubbeldam D., Calero S., Ellis D. E., Snurr R. Q. (2016). RASPA: molecular simulation software for adsorption and diffusion in flexible nanoporous materials. Mol. Simul..

[cit34] Manz T. A., Sholl D. S. (2010). Chemically meaningful atomic charges that reproduce the electrostatic potential in periodic and nonperiodic materials. J. Chem. Theory Comput..

[cit35] Maerzke K. A., Schultz N. E., Ross R. B., Siepmann J. I. (2009). TraPPE-UA force field for acrylates and Monte Carlo simulations for their mixtures with alkanes and alcohols. J. Phys. Chem. B.

[cit36] Rappé A. K., Casewit C. J., Colwell K., Goddard III W. A., Skiff W. M. (1992). UFF, a full periodic table force field for molecular mechanics and molecular dynamics simulations. J. Am. Chem. Soc..

[cit37] Kühne T. D., Iannuzzi M., Del Ben M., Rybkin V. V., Seewald P., Stein F., Laino T., Khaliullin R. Z., Schütt O., Schiffmann F. (2020). CP2K: An electronic structure and molecular dynamics software package-Quickstep: Efficient and accurate electronic structure calculations. J. Chem. Phys..

[cit38] Perdew J. P., Burke K., Ernzerhof M. (1996). Generalized gradient approximation made simple. Phys. Rev. Lett..

[cit39] Grimme S., Ehrlich S., Goerigk L. (2011). Effect of the damping function in dispersion corrected density functional theory. J. Comput. Chem..

[cit40] Stuke A., Todorović M., Rupp M., Kunkel C., Ghosh K., Himanen L., Rinke P. (2019). Chemical diversity in molecular orbital energy predictions with kernel ridge regression. J. Chem. Phys..

[cit41] Zhang L., Wang H., Car R., E W. (2021). Phase diagram of a deep potential water model. Phys. Rev. Lett..

