# Supplementary material for: Machine learning potential for modelling dynamic hydrogen bond networks in MOF MIL-120
Source: Chem Sci. 2026 Apr 2;17(20):10177–91. doi: 10.1039/d5sc09058j (PMC13071827; doi:10.1039/d5sc09058j)
Supplement: SC-017-D5SC09058J-s001 [file SC-017-D5SC09058J-s001.pdf]

Supplementary Information (SI) for Chemical Science.
This journal is © The Royal Society of Chemistry 2026

Supporting Information: Machine learning potential for modelling dynamic hydrogen bond networks in MOF MIL-120

Xin Jin, Yutao Li, Kelian Gaedecke, Xiaoqi Zhang, and Berend Smit*

Laboratory of Molecular Simulation (LSMO), Institut des Sciences et Ingénierie Chimiques, École Polytechnique Fédérale de Lausanne (EPFL), Switzerland

E-mail: berend.smit@epfl.ch

Contents

1 DFT functional benchmark **S-3**

2 MD simulation efficiency **S-4**

3 MLP Model comparison **S-4**

3.1 Molecular dynamics convergence S-9

3.2 GCMC simulation convergence S-10

1 DFT functional benchmark

To estimate the impact of different DFT functionals on MIL-120 adsorption calculations, we benchmarked the binding energy of the parallel adsorption configuration of CO_2 in Str2, as this represents the most favorable adsorption site. The functionals PBE, rPBE, revPBE, and B3LYP were selected, combined with either D3 or D3(BJ) van der Waals corrections. The results are summarized in the table :

Table S1: DFT binding energies for different functionals

**Functional**	**Binding energy (kJ/mol)**
PBE-D3	-46.95
PBE-D3(BJ)	-43.82
rPBE-D3	-25.15
rPBE-D3(BJ)	-22.00
revPBE-D3	-29.61
revPBE-D3(BJ)	-32.29
B3LYP-D3	-36.57
B3LYP-D3(BJ)	-37.11

The experimentally measured adsorption enthalpy of CO_2 in MIL−120 is $-40.2\,\mathrm{kJ\,mol^{-1}}$. Among the tested functionals, only PBE yields a binding energy for the most favorable adsorption site that exceeds this value. Consequently, the PBE functional with D3 correction was selected for the DFT calculations.

2 MD simulation efficiency

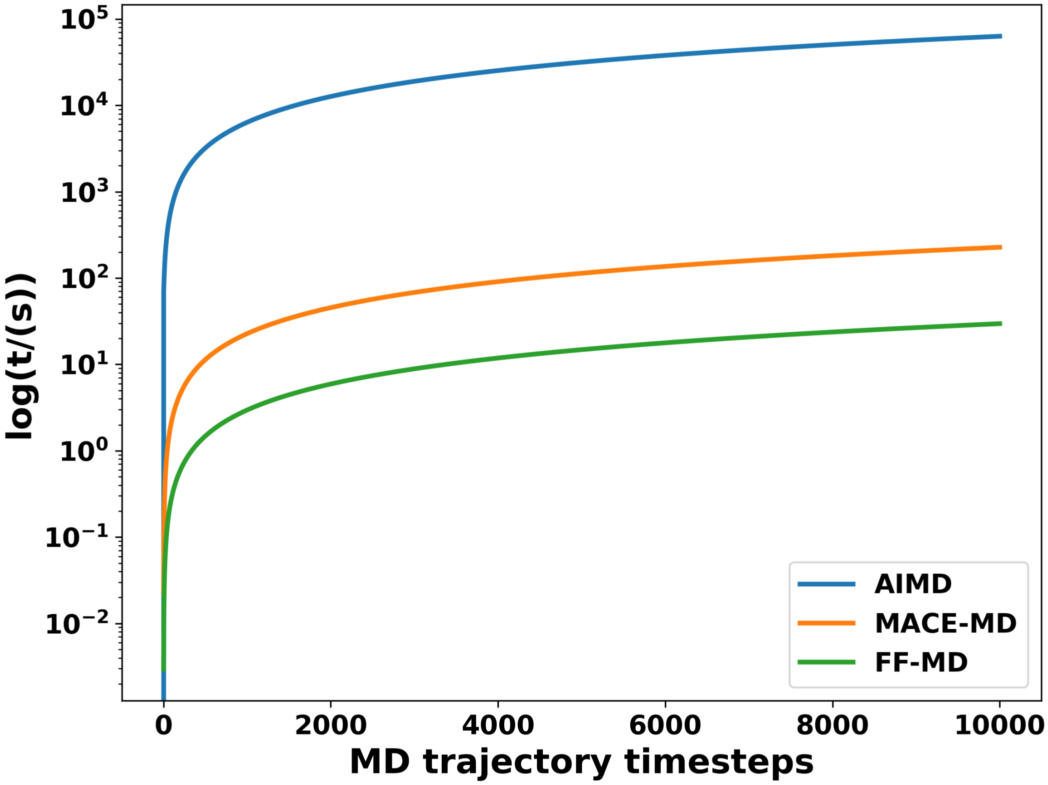

Figure S1: blackComparison between the MD simulation speed of different method. The AIMD simulations were performed using 72 MPI processes, whereas both FF-MD and MACE-MD simulations were carried out with a single MPI process. Even under parallel conditions, the computational speed of AIMD is still orders of magnitude slower than that of FF-MD and MACE-MD. Meanwhile, the computational efficiencies of MACE-MD and FF-MD are of the same order of magnitude, with MACE-MD being slightly slower than FF-MD.

3 MLP Model comparison

We have compared the performance of our fine-tuned model with the mace_mp_0b2-medium pretrained model and the mace-dac model on the same MIL-120 test set. As can be seen from S2, neither model achieves the level of accuracy required for this system to obtain meaningful thermodynamic properties. For example, the energy mean absolute error of the

mace_mp_0b2-medium model is around 0.6 meV/atom across different systems, while that of mace-dac is approximately 1.0 meV/atom. Here, the mean values of the two energy sets are shifted to the same level to avoid deviations caused by differences in the absolute energy values from different DFT simulation platform. Considering that the MIL-120 empty cell contains 160 atoms, such energy deviations would translate into an error bar as large as $\approx 20-40\,\mathrm{kJ\,mol^{-1}}$ for the predicted heat of adsorption, which is unacceptable. Furthermore, both atomic force MAEs exceed $100\,\mathrm{meV\,Å^{-1}}$, which would introduce a significant bias in the resulting MD trajectories. This highlights the necessity of our own fine-tuning workflow.

Table S2: Different MLP model performance on testing set

**Model**	**Qst (kJ/mol)**	**Empty MOF**		**MOF+CO_2**		**MOF+4CO_2**	
		MAE_E (meV/atom)	MAE_F (meV/Å)	MAE_E (meV/atom)	MAE_F (meV/Å)	MAE_E (meV/atom)	MAE_F (meV/Å)
`MACE-MP-0b2`	-28.55	0.55	109.18	0.60	105.82	0.70	103.54
`MACE-DAC`	2.46	1.03	196.13	1.23	195.29	0.97	190.92
`MACE-FT`	-41.90	0.07	14.59	0.07	13.33	0.09	14.50

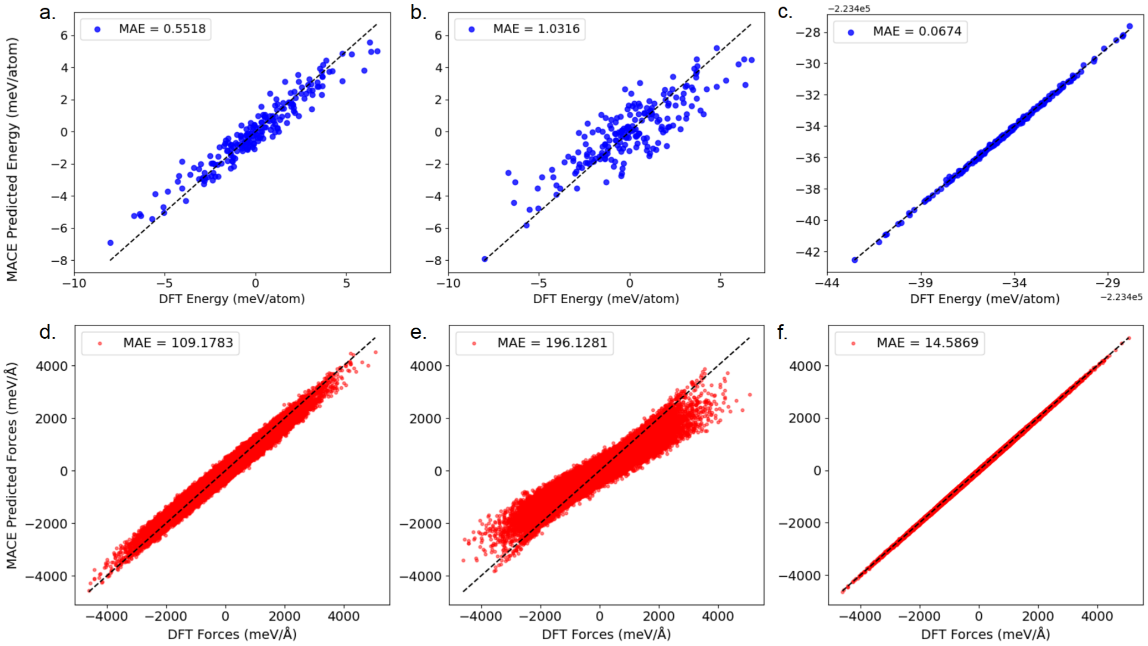

Figure S2: Comparison between different MLP model predictions and DFT results in terms of energy and interatomic force of the empty MOF framework system. (a)–(c) present the energy predictions of MACE-MP-0b2-medium, MACE-DAC, and MACE-FT (this work) on the same external test set, respectively; (d)–(f) present the corresponding interatomic force predictions.

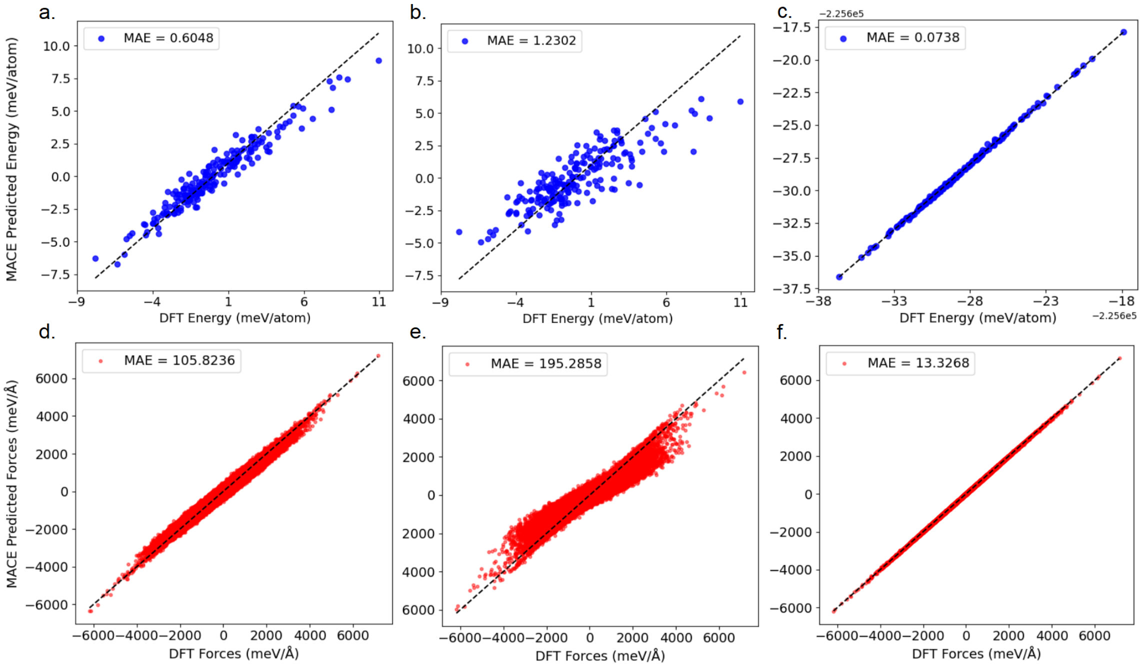

Figure S3: Comparison between different MLP model predictions and DFT results in terms of energy and interatomic force of the MOF with one CO_2 system. (a)–(c) present the energy predictions of MACE-MP-0b2-medium, MACE-DAC, and MACE-FT (this work) on the same external test set, respectively; (d)–(f) present the corresponding interatomic force predictions.

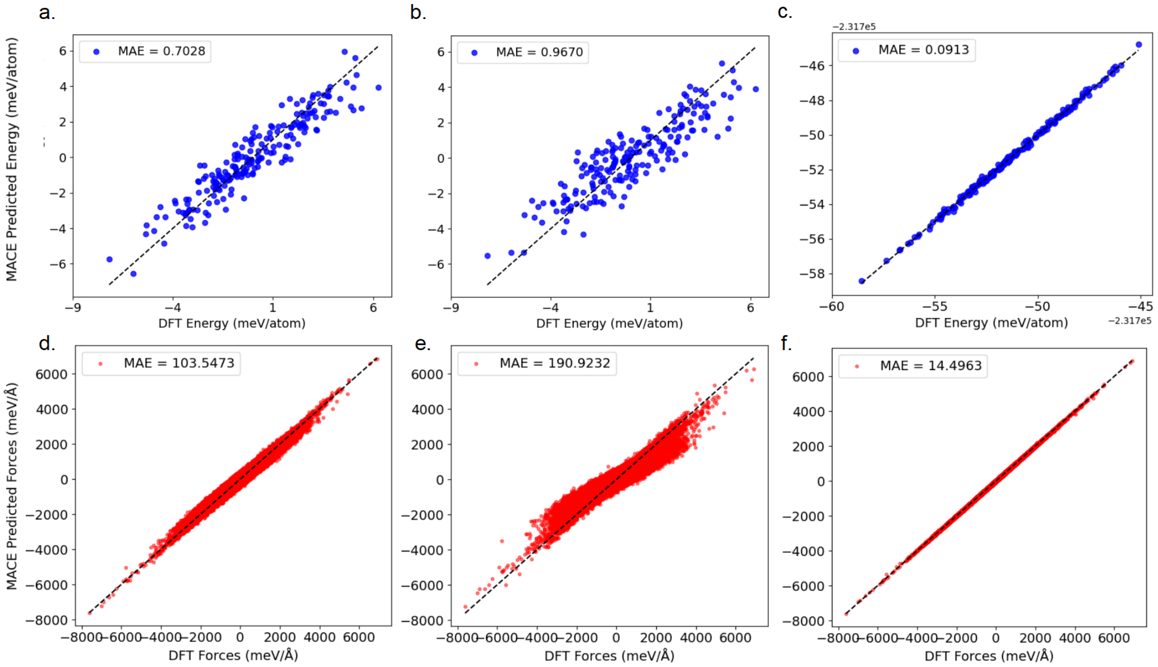

Figure S4: Comparison between different MLP model predictions and DFT results in terms of energy and interatomic force of MOF with 4 CO_2 system. (a)–(c) present the energy predictions of MACE-MP-0b2-medium, MACE-DAC, and MACE-FT (this work) on the same external test set, respectively; (d)–(f) present the corresponding interatomic force predictions.

3.1 Molecular dynamics convergence

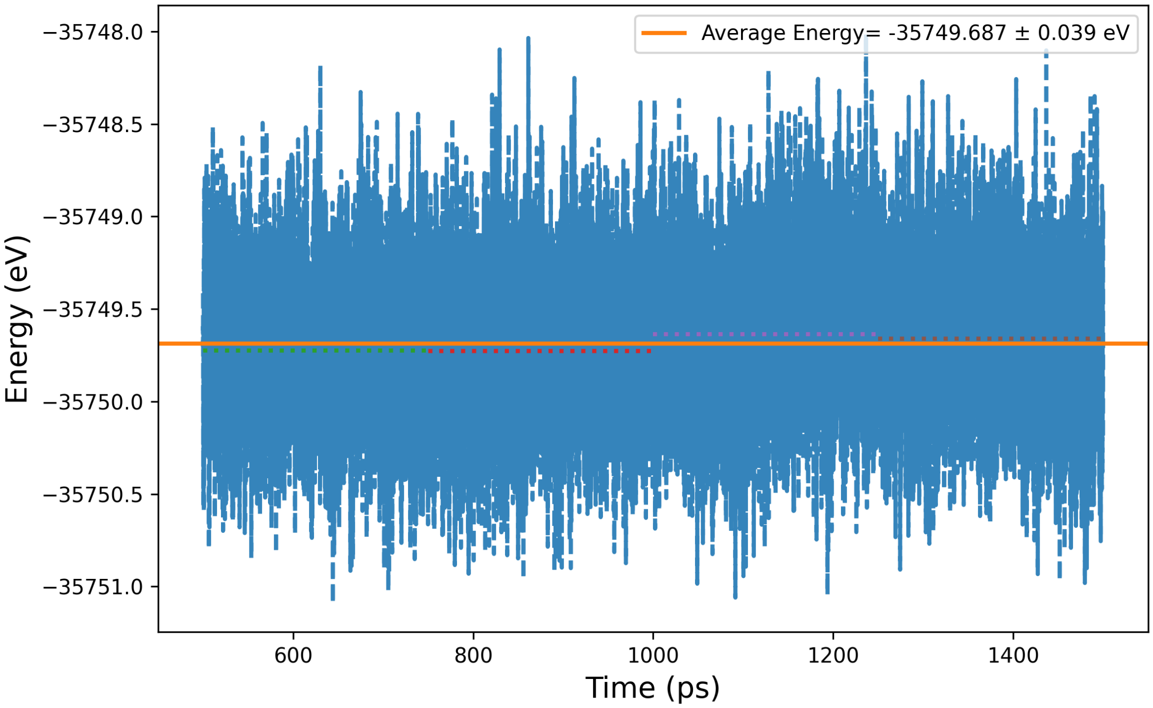

Figure S5: MACE-MD simulation of the empty MIL-120 structure. The dashed line denotes the average energy of the MIL-120 structure calculated over intervals of 0.25 ns, while the energy fluctuations within each block reflect variations among different structures.

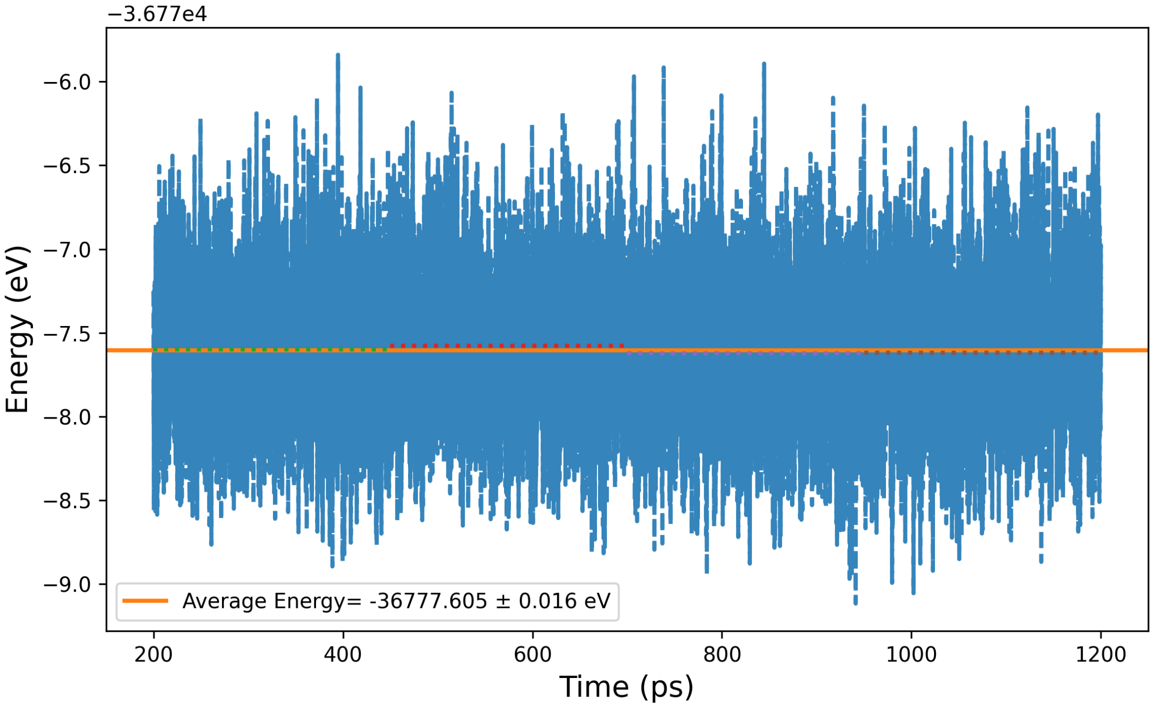

Figure S6: MACE-MD simulation of the MIL-120 with one CO_2 structure. The dashed line denotes the average energy of the loading structure calculated over intervals of 0.25 ns, while the energy fluctuations within each block reflect variations among different structures.

3.2 GCMC simulation convergence

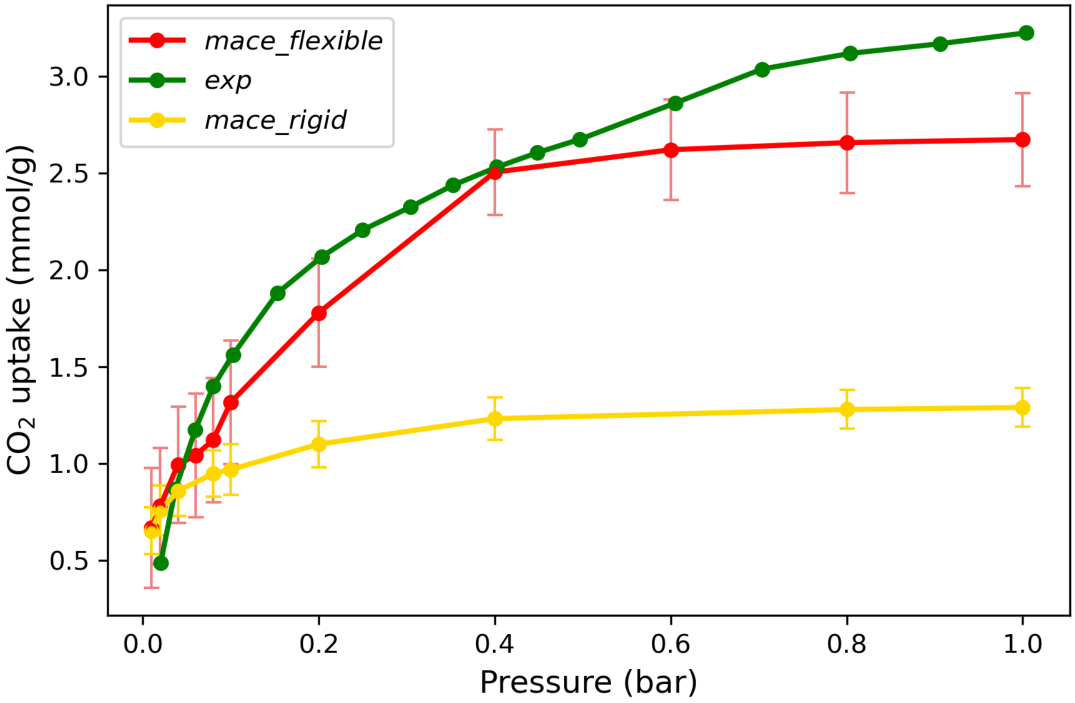

Figure S7: Comparison of the simulated and experimental CO_2 adsorption isotherms at 303 K. The green curve represents the experimental CO_2 adsorption isotherm. The red curve shows the result from this work using GCMC simulations with a fine-tuned MACE potential on a flexible framework. The yellow curve shows the result from this work using GCMC simulations with a fine-tuned MACE potential on a rigid framework.
